# Dissecting Monomer-Dimer Equilibrium of an RNase P Protein Provides Insight Into the Synergistic Flexibility of 5’ Leader Pre-tRNA Recognition

**DOI:** 10.3389/fmolb.2021.730274

**Published:** 2021-09-03

**Authors:** Danyun Zeng, Ainur Abzhanova, Benjamin P. Brown, Nicholas J. Reiter

**Affiliations:** ^1^ Department of Chemistry, Marquette University, Milwaukee, WI, United States; ^2^ Chemical and Physical Biology Program, Medical Scientist Training Program, Vanderbilt University, Nashville, TN, United States; ^3^ Center for Structural Biology, Vanderbilt University School of Medicine, Nashville, TN, United States

**Keywords:** ribonuclease P, solution NMR, tRNA processing, substrate recognition, diffusion coefficient, folding, binding, RNA

## Abstract

Ribonuclease P (RNase P) is a universal RNA-protein endonuclease that catalyzes 5’ precursor-tRNA (ptRNA) processing. The RNase P RNA plays the catalytic role in ptRNA processing; however, the RNase P protein is required for catalysis *in vivo* and interacts with the 5’ leader sequence. A single P RNA and a P protein form the functional RNase P holoenzyme yet dimeric forms of bacterial RNase P can interact with non-tRNA substrates and influence bacterial cell growth. Oligomeric forms of the P protein can also occur *in vitro* and occlude the 5’ leader ptRNA binding interface, presenting a challenge in accurately defining the substrate recognition properties. To overcome this, concentration and temperature dependent NMR studies were performed on a thermostable RNase P protein from *Thermatoga maritima*. NMR relaxation (R_1_, R_2_), heteronuclear NOE, and diffusion ordered spectroscopy (DOSY) experiments were analyzed, identifying a monomeric species through the determination of the diffusion coefficients (D) and rotational correlation times (τ_c_). Experimental diffusion coefficients and τ_c_ values for the predominant monomer (2.17 ± 0.36 * 10^−10^ m^2^/s, *τ*_c_ = 5.3 ns) or dimer (1.87 ± 0.40* 10^−10^ m^2^/s, *τ*_c_ = 9.7 ns) protein assemblies at 45°C correlate well with calculated diffusion coefficients derived from the crystallographic P protein structure (PDB 1NZ0). The identification of a monomeric P protein conformer from relaxation data and chemical shift information enabled us to gain novel insight into the structure of the P protein, highlighting a lack of structural convergence of the N-terminus (residues 1–14) in solution. We propose that the N-terminus of the bacterial P protein is partially disordered and adopts a stable conformation in the presence of RNA. In addition, we have determined the location of the 5’ leader RNA in solution and measured the affinity of the 5’ leader RNA–P protein interaction. We show that the monomer P protein interacts with RNA at the 5’ leader binding cleft that was previously identified using X-ray crystallography. Data support a model where N-terminal protein flexibility is stabilized by holoenzyme formation and helps to accommodate the 5’ leader region of ptRNA. Taken together, local structural changes of the P protein and the 5’ leader RNA provide a means to obtain optimal substrate alignment and activation of the RNase P holoenzyme.

## Introduction

Ribonuclease (RNase) P is an essential RNA processing enzyme involved in the 5’ endonucleolytic cleavage of precursor transfer RNA (ptRNA). It was one of the first identified examples of an RNA-catalytic reaction and exists as a multi-turnover RNA-based enzyme in bacteria, archaea, and eukaryotes (Guerrier-Takada et al., 1983). In bacteria, a large catalytic RNA (P RNA) and a small protein (P protein) component assemble as a holoenzyme complex to recognize and cleave ptRNA. Synergistic molecular recognition of ptRNA substrates by RNase P requires RNA-RNA shape complementarity, intermolecular base pairing, and stabilization of the 5’ leader single stranded RNA (ssRNA) region by the P protein component (Kazantsev and Pace 2006; Torres-Larios et al., 2006; Esakova and Krasilnikov 2010; Chan et al., 2013; Mondragon 2013; Klemm et al., 2016; Zhang and Ferré-DAmaré 2016; Gray and Gopalan 2020).

Structural studies of the RNase P holoenzyme with tRNA in bacteria and eukaryotes show how the RNA-protein complex functions as a single, monomeric assembly to selectively recognize ptRNA targets (Reiter et al., 2010; Lan et al., 2018; Wu et al., 2018). Interestingly, a cryo-EM structure of *Methanocaldococcus jannaschii* (Mja) RNase P reveals the components can be organized as a dimeric conformation for efficient catalysis (Wan et al., 2019). In all cases, the structures reveal conserved RNase P ribozyme features that are universal, such as a dual T-loop P RNA tertiary motif that recognizes the TΨC- D elbow region of tRNA and conserved P RNA nucleotides that comprise the ribozyme active site (Krasilnikov et al., 2003; Krasilnikov et al., 2004; Kazantsev et al., 2005; Torres-Larios et al., 2005). The collective X-ray and Cryo-EM determined structures suggest a largely pre-organized RNA active site, though a conserved and bulged uridine has been proposed to undergo a conserved dynamic motion that helps to position the substrate and trigger catalytic activation (Kaye et al., 2002; Christian et al., 2006; Reiter et al., 2010; Chen et al., 2011; Martin and Reiter 2017; Lan et al., 2018). These conserved structural and dynamic motions of the P RNA appear to be essential for accurate substrate recognition and the formation of the metallo-ribozyme active site.

In contrast to the P RNA, the RNase P protein has little or no structural similarity across all three domains but its structure is highly conserved in bacteria. The bacterial P protein is required for *in vivo* activity, binds the catalytic P RNA with nanomolar affinity utilizing its arginine-rich “RNR” motif, forms extensive interactions with the 5’ leader ptRNA region, increases catalytic efficiency by over two orders of magnitude, and facilitates product release (Peck-Miller and Altman 1991; Tallsjo and Kirsebom 1993; Talbot and Altman 1994; Crary et al., 1998; Kurz et al., 1998; Kurz and Fierke 2002; Buck et al., 2005a; Buck et al., 2005b; Sun et al., 2006; Koutmou et al., 2010b; Reiter et al., 2010; Sun et al., 2010; Koutmou et al., 2011; Reiter et al., 2012; Lin et al., 2016; Niland et al., 2017). The bacterial P protein is a highly stable, rigid scaffold that bind and stabilizes the P RNA and serves as a unique binding cleft for ssRNA. However, the bacterial P protein also contains some intrinsically disordered regions, especially within its N-terminus (Kirsebom 2007). This N-terminus (residues 1–21) plays a critical role in binding the P RNA as part of the holoenzyme complex and in optimally aligning the 5’ leader ptRNA substrate. We sought to better understand the structure and flexibility of the N-terminus of the P protein as well as define the 5’ leader RNA binding interaction using solution NMR spectroscopy.

High-resolution crystal structures and biochemical studies of the bacterial RNase P protein by itself have provided some insight into the ssRNA 5’ leader binding site, though no structure of an isolated P protein-5’ leader RNA complex has been determined to date (Stams et al., 1998; Spitzfaden et al., 2000; Kazantsev et al., 2003; Henkels et al., 2007; Ha et al., 2018). In addition, much less is known about how P protein flexibility contributes to 5’ leader binding and catalytic activation. We chose to explore the solution structure, protein flexibility, and 5’ leader binding properties of the RNase P protein from *Thermotoga maritima* because extensive structure, biochemical analyses, and small molecule screening studies have been performed on this system (Paul et al., 2001; Krivenko et al., 2002; Buck et al., 2005a; Torres-Larios et al., 2005; Reiter et al., 2010; Reiter et al., 2012; Madrigal-Carrillo et al., 2019). The high-resolution structure of the *Thermotoga maritima* RNase P protein, hence termed P protein, crystallized as a tetramer and its oligomeric state occluded the 5’ leader binding through lattice contacts at the P protein’s N-terminus (Kazantsev et al., 2003). This propensity towards oligomerization makes structural analysis of the 5’ leader- P protein interface recalcitrant and intractable to crystallographic methods.

Here, we describe an NMR-based strategy to overcome oligomerization of the P protein from *Thermotoga maritima* and directly monitor two protein conformers that coexist in solution. The identification of an NMR-dervied monomeric model emphasizes the lack of structure within the N-terminus and allowed us to define the 5’ leader RNA binding properties of the bacterial P protein.

## Materials and Methods

### Sample Preparation

The ^15^N, ^13^C-labeled *T. maritima* RNase P protein sample was prepared following the previous protocol (Zeng et al., 2018). A pGEX4Ta vector containing the *rnpA* gene from *T. maritima* and an N-terminal glutathione S-transferase (GST) fusion protein was transformed and expressed in the BL21 Gold *Escherichia coli* cell strain and cultured in M9 minimal media at 303 K supplemented with ^15^N ammonium chloride and/or ^13^C glucose. After lysis sonication in the presence of cOmplete™ protease inhibitor cocktail (Roche), lysate was separated by centrifugation (28,000 g), filtered, and treated with 800 NIH units of thrombin per 40 ml to remove the GST tag from RNase P protein. A denaturation−renaturation purification strategy was applied (Paul et al., 2001; Krivenko et al., 2002; Buck et al., 2005a; Buck et al., 2005b; Reiter et al., 2012). The thrombin-treated lysate was combined with denaturation buffer (50 mM Tris-HCl pH7.5, 4 mM EDTA, 8 M urea) to a final concentration of 5 M urea, and was subject to 15S cation exchange chromatography. Fractions containing the denatured protein were subsequently dialyzed against refolding buffer (50 mM Tris-HCl pH 7.5, 0.2 mM EDTA, 1 M NaCl) for 1–2 days, upon which the sample was diluted and subject to a second 15S cation exchange column under the identical condition excluding urea. Purified ^15^N, ^13^C-labeled RNase P protein fractions were collected, concentrated, and dialyzed against a buffer of 50 mM Tris-HCl pH 7.5, 0.2 mM EDTA.

### NMR Spectroscopy

All NMR experiments were conducted using either low (153 μM) or high (466 μM) ^15^N, ^13^C RNase P protein concentrations in 20 mM sodium phosphate pH 6.0, 80 mM NaCl, 50 μM 4, 4-dimethyl-4-silapentane-sulfonate (DSS) at 318 K with 10% (v/v) D_2_O in a 3 mm Norell^®^ select series NMR tube. NMR spectra were acquired on Bruker Avance-III 600 and 800 MHz and 900 MHz spectrometers, equipped with cryogenic probes, as well as Varian VNMRS 600 and 800 MHz spectrometer equipped with a cryogenic probe. NMR spectra were processed with Topspin 3.5.7 (Bruker Inc.) and NMRPipe (Delaglio et al., 1995), and analyzed with NMRViewJ (One Moon Scientific, Inc.) and CARA (Keller 2004). ^1^H chemical shifts were referenced with respect to internal DSS, and ^13^C and ^15^N chemical shifts were referenced indirectly using nuclei-specific gyromagnetic ratios (Wishart et al., 1995).

### NMR Assignments of the P Protein Backbone According to Different Concentrations and Temperatures

NMR chemical shift assignments of the protein backbone were determined on separate samples with protein concentrations of 466 and 153 μM, respectively. At the higher protein concentration, backbone assignments were derived from ^1^H, ^15^N-heteronuclear single quantum coherence (HSQC) and a set of traditional triple-resonance experiments, including HNCO, HNCA, HN(CO)CA, HNCACB, CBCA(CO)NH, and HN(CA)CO. In addition, backbone and side-chain assignments were carried out using a three-dimensional ^15^N-NOESY-HSQC experiment (120 ms mixing time). At the lower protein concentration, backbone assignments were confirmed with ^1^H, ^15^N-HSQC, HNCO, HNCA, and HNCACB, comparing and referencing to the assignments of the higher concentration sample. The chemical shift assignments for the 466 μM and the 153 μM sample were deposited to the biological magnetic resonance bank (BMRB) as accession numbers 27307 and 51021, respectively. To investigate the temperature dependence of the backbone assignments of P protein (153 μM), a set of ^1^H, ^15^N-HSQC spectra were acquired with identical parameters at 35 (308), 45 (318), 55 (328), and 65°C (338 K). Additional parameters and data processing, including window function, base-line correction, and linear prediction were identical for all ^1^H, ^15^N-HSQC spectra.

### Diffusion Measurements of RNase P Protein

Two ^13^C/^15^N-labeled protein samples with concentrations of 153 and 466 μM were prepared for high-resolution DOSY (diffusion-ordered NMR spectroscopy) experiments. 2D DOSY-^1^H, ^15^N-HSQC experiments were performed on a Varian VNMRS 800 MHz spectrometer with a diffusion delay (Δ) of 70 ms and gradient width (*δ*) of 3 ms at 318 K. Six different gradient strengths (0.9, 9.7, 18.6, 27.4, 36.2, and 45.1 Gauss/cm) were used to measure diffusion rate of the sample with higher concentration; while 8 gradient strengths (0.88, 7.24, 13.6, 20.0, 26.3, 32.7, 39.1, and 45.4 Gauss/cm) were applied to the low concentration sample. Each experiment was acquired with a data matrix of 168 (t_1_, ^15^N) × 2048 (t_2_, ^1^H) complex points and 64 scans. NMRPipe and NMRViewJ were used for data processing and analysis, respectively. The exact centers of the cross peaks for the selected resonances were identified and the fitting of the results and error were propagated in the analysis of signal decay curves. Reference diffusion coefficients for the RNase P monomer, dimer, and tetramer species were calculated from the high-resolution crystal structure (PDB: 1NZ0) using HYDROPRO and HYDRONMR (García de la Torre et al., 2000; Ortega et al., 2011). Molecule A, A/C, and A-D of PDB 1NZ0 were used for simulations of the monomer, dimer, and tetramer, respectively.

### Measurement of Relaxation Parameters

Relaxation parameters were measured at 318 K of the 466 μM P protein on a Bruker Avance-III 800 MHz spectrometer. For R_1_, five relaxation time points were collected at 100, 200, 400, 600, and 1,000 ms. For R_2_, five relaxation time points were collected at 17, 34, 51, 85, and 119 ms. For R_1_ and R_2_ measurements, a recycle delay of 1 s was used between transitions. Errors were estimated by duplicate measurements using the shortest and longest relaxation time. For heteronuclear NOE measurements, the steady-state ^1^H saturation time was 5 s, a recycle delay of 5 s was implemented in the reference experiment, and both reference and NOE measurements were repeated in duplicate. All spectra of relaxation measurements were collected with the data matrix of 300 (t_1_, ^15^N) × 1,024 (t_2_, ^1^H) points and 32 scans. NMRPipe was used for data processing and data were evaluated using the rate analysis module of NMRViewJ (Johnson and Blevins 1994; Delaglio et al., 1995). To estimate rotational correlation time from R_1_ and R_2_ values, calculation were made by performing Eq. 1 (Kay et al., 1989):

$$\tau_{c}\approx \frac{1}{4{\text{πν}}_{\text{N}}}\sqrt{6\frac{R_{2}}{R_{1}}-7}$$ (1)

where ν_N_ is the ^15^N resonance frequency in Hz.

### *T. maritima* RNase P Model Prediction Based on NMR Chemical Shifts

Two sets of NMR chemical shifts were extracted from the high and low protein concentration samples (BMRB #27307 and #51021) and models were generated using chemical-shift (CS-) Rosetta predictive modeling as described elsewhere (Shen et al., 2008; Shen and Bax 2013). The identical number of chemical shift assignments from the “low” and “high” concentration samples were imported to the CS-Rosetta web server to assess predictive modeling results in a direct, comparative manner. NMR chemical shift information was the only experimental data incorporated into model prediction calculations. A total of 3,000 structural models were generated for each set (low and high P protein concentration) and the top 10 best scoring models were selected. Visualization and analysis of predictive models were performed in Molmol (Koradi et al., 1996).

### The Interaction Between RNase P Protein and 5’ Leader RNA

NMR titrations investigated the binding process between the RNase P protein and the ptRNA 5’ leader. A 7-mer 5’ leader sequence of ptRNA (AAGGCGU) was purchased from Dharmacon Co. with purity >95%. Both protein and ptRNA leader were pre-equilibrated in the identical NMR buffer as described above. During titrations, the leader RNA was gradually added with increased molar ratios of 0.2:1, 0.4:1, 1:1, and 2:1 to the 153 μM protein sample. At each molar ratio, a ^1^H, ^15^N-HSQC spectrum was acquired at 318 K. All data were processed by NMRpipe and analyzed by NMRviewJ. The analysis of amide chemical shift changes (Δδ) were calculated following Eq. 2 (Schumann et al., 2007; Collier et al., 2014):

$$\text{Δδ}\left(\text{ppm}\right)=\sqrt{\text{Δδ}\left(1\text{H}\right)2+(0.152\text{Δδ}\left(15\text{N}\right))2}$$ (2)

Δδs of the peaks at the highest leader RNA concentration (5’ leader/protein molar ratio = 2:1) were used for chemical shift perturbation (CSP) analysis. Dissociation constants (K_D_) for each shifting peak were calculated by fitting the data to Eq. 3:

$$\Delta \text{δ}\left(\text{ppm}\right)=\Delta_{\text{max}}\frac{\left({\text{K}}_{\text{D}}+\left[\text{L}\right]+\left[\text{P}\right]\right)-\sqrt{{\left({\text{K}}_{\text{D}}+\left[\text{L}\right]+\left[P\right]\right)}^{2}-4\;\left[\text{P}\right]\left[\text{L}\right]}}{\;2\left[\text{P}\right]}$$ (3)

where ∆δ(ppm) is the amide chemical shift changes, Δ_max_ the maximum amide chemical shift changes, [P] and [L] the protein and ligand concentrations, respectively. Only defined monomeric chemical shift assignments were used for the K_D_ calculation. However, the CSP analysis included monomer-only peaks and identified monomer-dimer assigned peaks. In the 153 μM P protein sample, dimer-only peaks undergo chemical shift changes upon RNA binding but were too weak and unreliable to be included in the analysis.

### Molecular Docking of RNase P Protein and 5’ tRNA Leader

HADDOCK was used to predict the structure of the RNase P protein-5’ ptRNA leader complex, based on the CSP results of NMR titrations (Dominguez et al., 2003). Certain residues which have ∆δs larger than ∆δ_average_ + STD_∆δ_ were set as active residues, while amides having ∆δs between ∆δ_average_ and ∆δ_average_ + STD_∆δ_ were set as passive residues. The initial structure of the P protein was the representative structure from the CS-Rosetta calculated ensemble that incorporated the NMR peak assignments at low protein concentration. The initial model of the 7-mer ptRNA leader was generated in Coot (Emsley and Cowtan 2004). The docking calculations were performed on the HADDOCK web server; generating 127 predicted models using standard HADDOCK settings.

## Results

### Concentration Dependent Equilibrium of *T. maritima* RNase P Protein in Solution

**FIGURE 1:**
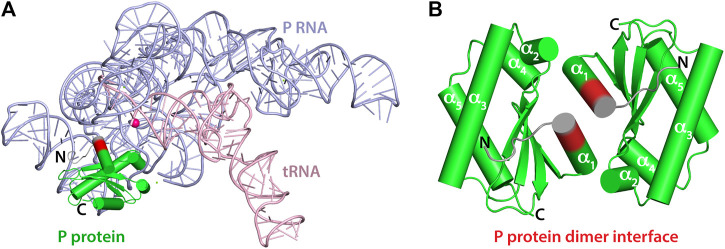
Crystal structures of *T. maritima* RNase P components. **(A)** Crystal structure of the holoenzyme in complex with tRNA (PDB 3Q1Q). RNase P RNA component is shown in light blue, the P protein component in green, and tRNA in light pink. The enzyme active site is denoted by the location of a catalytic magnesium ion (magenta sphere). RNAs are represented as loops (backbones) and sticks (nucleobases) and the P protein is represented as cylinders (α-helix) and arrows (β-sheets). Protein residues 14–17 are highlighted in red to indicate the location of the dimerization interface of the P protein alone (PDB 1NZ0). Residues 8–21 play a critical role in binding the P RNA as part of the holoenzyme complex and in optimally aligning the 5’ leader ptRNA substrate. **(B)** 1.2 Å resolution crystal structure of the P protein shown as a dimer, with molecules A and C (PDB 1NZ0). Protein residues 14–17 are highlighted in red and the N-termini in colored grey. Residues 1–15 are oriented differently between **A** and **B**.

**FIGURE 2:**
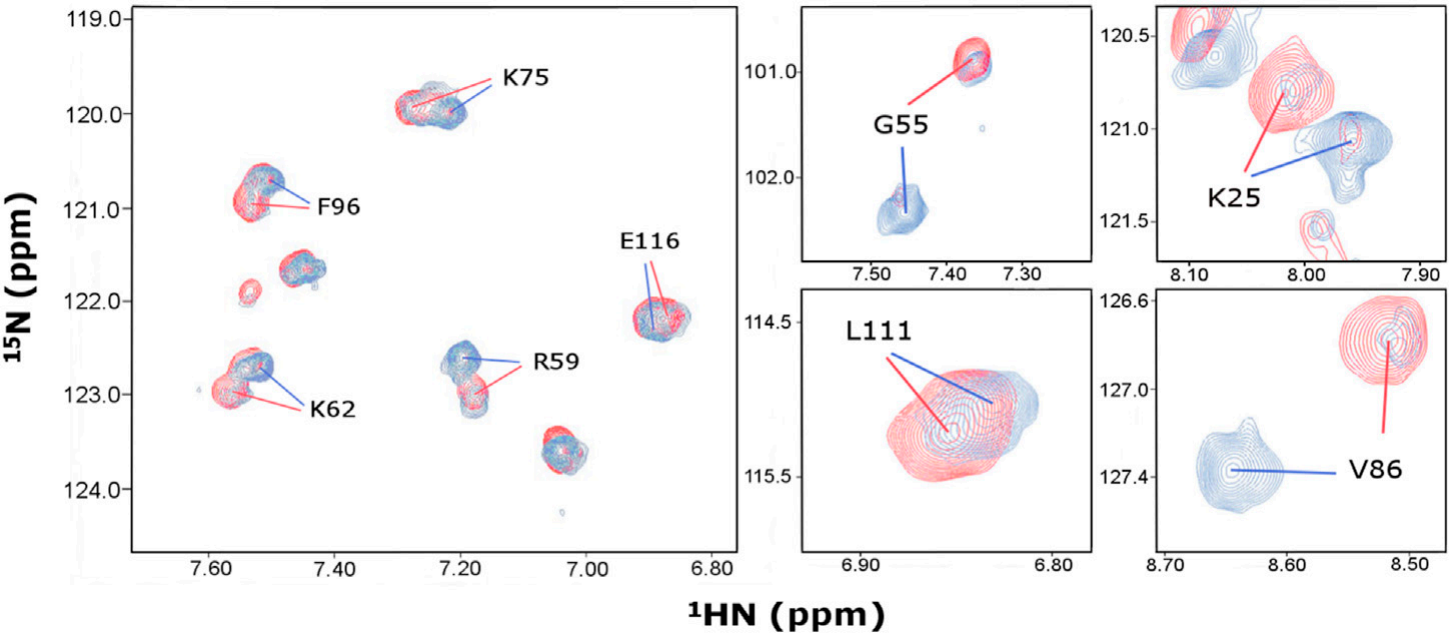
Identification of distinct chemical shift assignments at 466 and 153 μM P protein concentrations. Overlay of ^1^H, ^15^N-HSQC spectra acquired on samples containing 466 μM (red) and 153 μM (blue) P protein concentrations under identical buffer conditions at 318K. Representative regions of the spectra are shown, revealing slow exchange between monomer and dimer conformers in solution. Amino acid *single letter code* and *T. maritima* RNase P protein *numbering* corresponds to UniProtKB–Q9X1H4.

The functional *T. maritima* ribonuclease P holoenzyme structure contains single RNA (ribozyme) and protein components, yet the large 110 kDa P RNA-only crystallizes as a dimer and the small 14 kDa P protein-only crystal structure (PDB-1NZ0) contains two dimers within its asymmetric unit (Figure 1) (Paul et al., 2001; Kazantsev et al., 2003; Torres-Larios et al., 2005; Reiter et al., 2012). The P protein dimer interface in the crystal structure obscures the 5’ leader RNA binding track (Kazantsev et al., 2003) and this is problematic in characterizing the 5’ leader pre-tRNA substrate-protein interaction. Preliminary NMR experiments reveal that highly purified *T. maritima* RNase P protein, hence termed P protein, persists as a dimer at 25°C but is a heterogeneous species in solution. Thus, oligomerization is not merely an artifact of crystallization. To define the NMR assignments of an RNA binding competent P protein, ^1^H, ^15^N-HSQC spectra at various temperatures and on samples containing protein concentrations in the range of 153–466 μM were collected and analyzed. A series of triple-resonance experiments at both high (466 μM) and low (153 μM) protein concentrations were also collected and analyzed at 45°C. At P protein concentrations of 153 and 466 μM, there are always 2 conformations in solution (Figure 2, Supplementary Figure S1). The analysis of various concentration and temperature spectra enabled the identification and assignment of two sets of amide peaks that correspond to two conformations in solution (BMRB #27307 and #51021).

When comparing P protein low (153 μM) and high (466 μM) concentrations at 45°C, we observed overall peak intensity changes between the two conformations depending upon the protein concentrations (Figure 2). This suggests a concentration-dependent shift in the equilibrium of the two species in solution. Between 35 to 65°C, similar temperature dependent chemical shifts were observed for both samples but no significant changes occurred in the relative peak intensities for the two conformations (Supplementary Figure S2). This suggests that both conformations are stable over a wide temperature range and that the oligomeric equilibrium of the P protein is concentration dependent and temperature-independent in the range of 35–65°C.

We report high confidence backbone assignments at high (96.5% amides, 99.1% for all C_α_ and C_β_, and 97.4% C’ resonances) and low P protein concentrations (93.0% amides, 90.6% for C_α_, 70.7% for C_β_, and 87.2% C’ resonances). Nearly 50% of all residues have different chemical shift assignments in two conformations of the ^1^H, ^15^N-HSQC, thus facilitating the accurate analysis of the distinct conformations of the P protein (Supplementary Table S1, BMRB #27307 and #51021).

### Identification of a Monomer and Dimer P Protein

To define oligomerization status of the distinct conformations, 2D-DOSY spectra were acquired at low (153 μM) and high (466 μM) P protein concentrations (Table 1). Analysis was performed on 48 residues that contained distinct chemical shifts for the two conformations in the ^1^H, ^15^N-HSQC spectra. Peak intensities of the dominant signal at low or high P protein concentrations were identified and selected for signal decay curve analyses from DOSY experiments. The average diffusion coefficients at 45°C were determined from the analysis of the selected peak intensities against gradient field strengths (Table 1). Experimental diffusion coefficient values of the low (153 μM) concentration conformer (D_low_ = (2.17 ± 0.36)*10^–10^ m^2^/s) and the high (466 μM) concentration conformer (D_high_ = (1.87 ± 0.40)*10^–10^ m^2^/s) are consistent with HYDROPRO and HYDRONMR software-simulated diffusion coefficients of a monomer (D ∼ 1.96 *10^–10^ m^2^/s) and dimer (D ∼ 1.54 *10^–10^ m^2^/s) P protein conformation (Table 1). The calculated, reference diffusion coefficients from HYDROPRO and HYDRONMR were derived from the high-resolution crystal structure PDB-1NZ0. A value difference of 0.3 for the experimental diffusion data (D ∼ 2.17 vs. 1.87) compared to the 0.42 value difference in the calculated values (D ∼ 1.96 vs. 1.54) reflect the fact that both the low and high concentration samples contain mixed monomer/dimer populations. While there is no evidence of higher order oligomerization, the monomeric form predominates at low protein concentration and the dimeric form primarily occurs at higher concentrations. The resolution and calculated error in the NMR-derived diffusion coefficients likely reflect the mixed populations present at both concentrations. Nonetheless, this comparative diffusion coefficient analysis identifies that the assigned resonances in the low (153 μM) and high (466 μM) concentration P protein samples correspond primarily to the monomeric and dimeric species, respectively.

**TABLE 1:** Experimental and calculated diffusion coefficients (D, ^*^10^-10^ m^2^/s) of *T. maritima* RNase P protein.

Method	Conformation					
DOSY	153 μM concentration			466 μM concentration		
	**2.17 ± 0.36**			**1.87 ± 0.40**		
		Monomer		Dimer		Tetramer	
HYDROPRO	**1.96**		**1.55**		**1.08**	
HYDRONMR	**1.93**		**1.53**		**1.10**	

**FIGURE 3:**
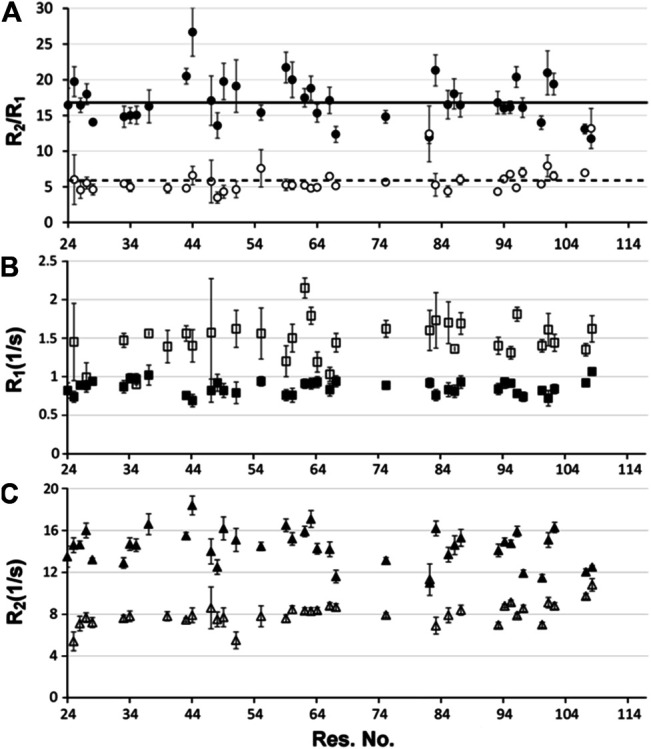
NMR relaxation analysis **(A)** R_2_/R_1_ ratios, **(B)** R_1_ and **(C)** R_2_ of distinct *T. maritima* RNase P protein conformers. The dominant P protein conformer at 466 μM is shown in filled circles, rectangles, and triangles in the three plots, respectively; while the minor conformer is presented as the open/white filled symbols. The R_2_/R_1_ ratio (A), as well as individual R_1_ (B) and R_2_ (C) relaxation experiments were performed on a 466 uM P protein sample at 318K. For each data set, errors represent multiple ^15^N T_2_ and T_1_ relaxation experiments in addition to the uncertainty of the exponential fit. The average R_1_, R_2_, and R_2_/R_1_ ratios of the dominant and minor P protein conformations are indicated as solid and dashed lines, respectively.

Verification of the identified monomer/dimer species was also determined through the analysis of the R_2_/R_1_ relaxation data. Despite heterogeneous sample conditions, 48 chemical shifts were extracted from two sets of residues in the spectra. One set of analyzed peaks was representative of the dominant conformer at the high concentration (466 μM) and the other set of peaks was representative of a minor conformer that is also present at 466 μM P protein. The observed minor conformer chemical shifts at 466 μM are identical to the dominant conformer at 153 μM P protein concentration. R_1_ and R_2_ rates were determined for the selected residues of the minor and dominant P protein conformers and their average R_2_/R_1_ ratios identified as 5.95 and 17.04, respectively, (Figures 3A–C). R_2_ values were considerably higher for peaks arising from the dominant conformer in comparison to peaks from the minor conformer (Figures 3A,C). In addition, rotational correlation times (τ_c_) (Kay et al., 1989) were determined from R_2_/R_1_ ratio values, revealing τ_c,low_ and τ_c,high_ values of approximately 5.3 and 9.7 ns, respectively. These parameters support a monomer–dimer equilibrium model, where the monomer conformer (τ_c, monomer_ ∼ 5.3 ns) predominates at ∼150 μM and the dimer species (τ_c, dimer_ ∼9.7 ns) predominates at higher P protein concentrations. The estimated τ_c_ values are substantially smaller than a typical 14 or 28 kDa idealized spherical protein at 25°C, but are consistent with the estimated empirical range of correlation times at 45°C (http://nickanthis.com/tools/tau). Thus, analysis of two independent NMR methodologies (DOSY and NMR relaxation) both validate that monomer and dimer P protein assembles coexist in solution. The monomeric conformation predominates at 153 μM P protein concentrations whereas the dimer species predominates at higher (466 μM P protein) concentrations.

### Predictive Modeling of the 2 Conformations

**FIGURE 4:**
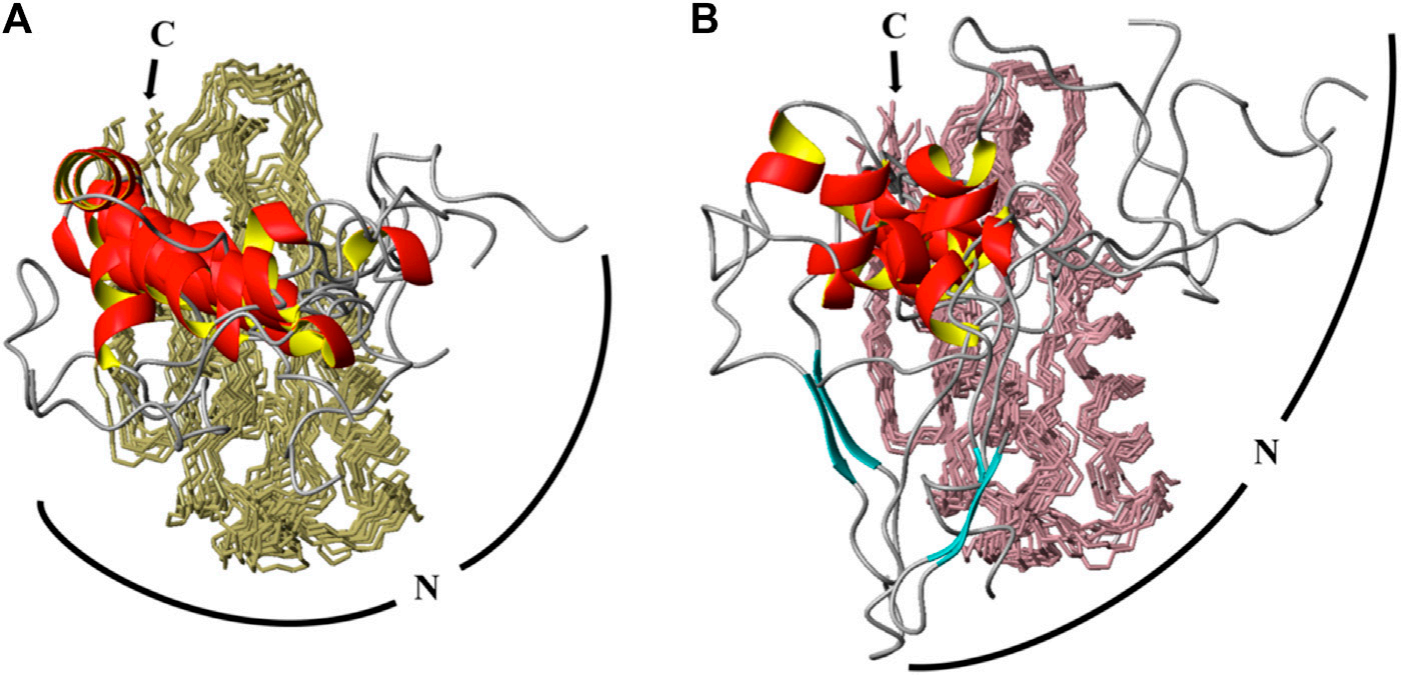
CS-Rosetta calculated ensembles of *T. maritima* P protein at high (466 μM) and low (153 μM) concentrations. The same number of chemical shifts arising from the same residues (distributed equally) for identified monomer and dimer conformations were incorporated into CS-Rosetta calculations. The top 10 scored models of each conformation are overlaid in **A** (at 466 μM) and **B** (at 153 μM). In both ensembles, 10 structures models are superposed from residue 24 to 117, with backbone regions colored olive (**A**) and light pink (**B**). The N-terminus region (residues 1–23) are presented as loop (gray coil-coiled) and ribbons (red helix) segments. The C-terminus (C) is labeled and a curved line of the N-terminus (N) is included to highlight the predicted amplitudes and flexibility observed in the dimer (**A**) and monomer (**B**) derived ensembles.

The two sets of backbone NMR assignments (HN, C_α_, and C’) based upon BMRB entries #27307 and #51021 were input into CS-Rosetta web server to generate models of the monomer and dimer forms of the P protein in solution. To inspect the structural differences caused only by chemical shifts, the same numbers of chemical shifts from exactly the same assigned resonances were implemented for CS-Rosetta calculations. The ensemble models converge well and reflect the dimeric and monomeric forms of the P protein in solution (Figures 4A,B, respectively). All ensembles include the 10 lowest-energy models and both sets of predicted structures contain near identical backbone folds that are consistent with the crystal structure (PDB: 1NZ0). However, whereas the backbone RMSD of residues 24–117 of the monomer and dimer species compared to PDB-1NZ0 are 0.97 Å and 1.19 Å, respectively, both models show a higher degree of flexibility at the N-terminus (residues 1–23). Both monomeric and dimeric conformers exhibit a lack of structural convergence within the first 23 residues at N terminal of the P protein, with a higher degree of structural variability observed in the monomeric-derived ensemble (Figure 4B).

A comparison of NOEs within the N-terminus (residues 8–12, 18–22) and a defined *α*-helical region (residues 59–68) reflect the extent or lack of structural convergence observed in CS-Rosetta models (Supplementary Figure S3). Poorly ordered regions at the N-terminus (residues 8–12) reveal only a few HN-HN (i,i+1) NOEs, a partially structured region that contains *α*-helical character (residues 18–22, α_1_ helix) reveals sequential (i, i+1) and some medium range NOEs, and a highly ordered region (residues 59–68, α_3_ helix) shows HN-HN (i, i+2), H_α_-HN (i, i+3), and H_α_-HN (i ,i+4) medium and long range NOE connectivities. The representative NOEs identified in ^15^N NOESY-HSQC spectrum highlight the lack of structural convergence in the N-terminus observed in the CS-Rosetta model.

Thus, comparative modeling and analysis of NOE data reveal that the N-terminal P protein is more flexible than other regions of the P protein. The CS-Rosetta data also suggest that the N-terminal region of the monomer conformation is more flexible than the dimer conformer despite using the exact same number of chemical shifts from the same assigned resonances. The alpha1 helix (α1) extends from residue 14–23 in the dimeric assigned CS-Rosetta ensemble with a backbone RMSD of 0.38 Å relative to PDB 1NZ0 (Figure 4B). This implies that the dimerization interface in solution is similar to the X-ray structure (PDB 1NZ0) (Figure 1B, Supplementary Figures S4, S5). In contrast, the monomer assigned CS-Rosetta models reveals a truncated α1 helix (residues 17–23) that appears intrinsically disordered (RMSD ∼1.56 Å) relative to 1NZ0. Taken together, this NMR-based data supports a structural mechanism whereby the N-terminal region (residues 1–12) becomes stabilized by the P RNA in the RNase P holoenzyme crystal structure (Reiter et al., 2010) and the transient structure of the N-terminal helix (residues 13–23) helps to optimally align the 5’ leader ssRNA substrate region (Niranjanakumari et al., 1998; Zahler et al., 2003; Buck et al., 2005a; Buck et al., 2005b; Sun et al., 2006; Niranjanakumari et al., 2007; Koutmou et al., 2010b; Reiter et al., 2012; Lin et al., 2016; Niland et al., 2017).

### The Binding Mode of P Protein and 5’ Pre-tRNA Leader

To understand the mechanism of RNase P holoenzyme activation and ptRNA cleavage, it is essential to study the structure and binding interaction between RNase P protein and 5’ ptRNA leader. We chose to work with RNase P from *Thermotoga maritima* because extensive structure, biochemical analyses, and small molecule screening studies have been performed on the RNase P holoenzyme (Paul et al., 2001; Buck et al., 2005a; Torres-Larios et al., 2005; Reiter et al., 2010; Reiter et al., 2012; Madrigal-Carrillo et al., 2019). We determined that the functionally relevant P protein monomer predominates at low (153 μM) concentrations, enabling NMR titrations to probe the binding interactions of the P protein and 5’ leader RNA. Chemical shift perturbation (CSP) analysis was performed when the 5’ leader RNA (5′- AAGGCGU-3′) was titrated into the P protein to a ratio of 2:1. Higher RNA:protein titration ratios were attempted >4:1 yet were followed by rapid sample precipitation, prohibiting the accurate collection of reliable chemical shift information. Nonetheless, CSP analysis of the protein-RNA titration was monitored via ^1^H, ^15^N-HSQC experiments, focusing on 57 assignments attributed to the monomer, 28 peaks corresponding to both monomer and dimer conformers, and 8 resonances attributed solely to the dimer species (Supplementary Figure S6). Dimer-identified peaks are minor at the 153 μM P protein concentrations (Figure 2); yet detectable minor chemical shift differences (Δδ) upon ligand binding were observed for both monomer and dimer species (Supplementary Figure S7). Specifically, residues Q28 and F82 represent dimer peaks that were shifted upon RNA titration at 153 μM P protein concentrations. Both Q28 and F82 HN resonances are structurally accessible to bind RNA in the monomer and dimer conformers yet only Q28 exists at a functionally relevant RNA binding interface. While it is possible that further RNA addition may shift the equilibrium from a dimer species towards the monomeric conformation, it is unclear from the available NMR data whether RNA binding substantially alters the dimer-monomer conformational equilibrium.

**FIGURE 5:**
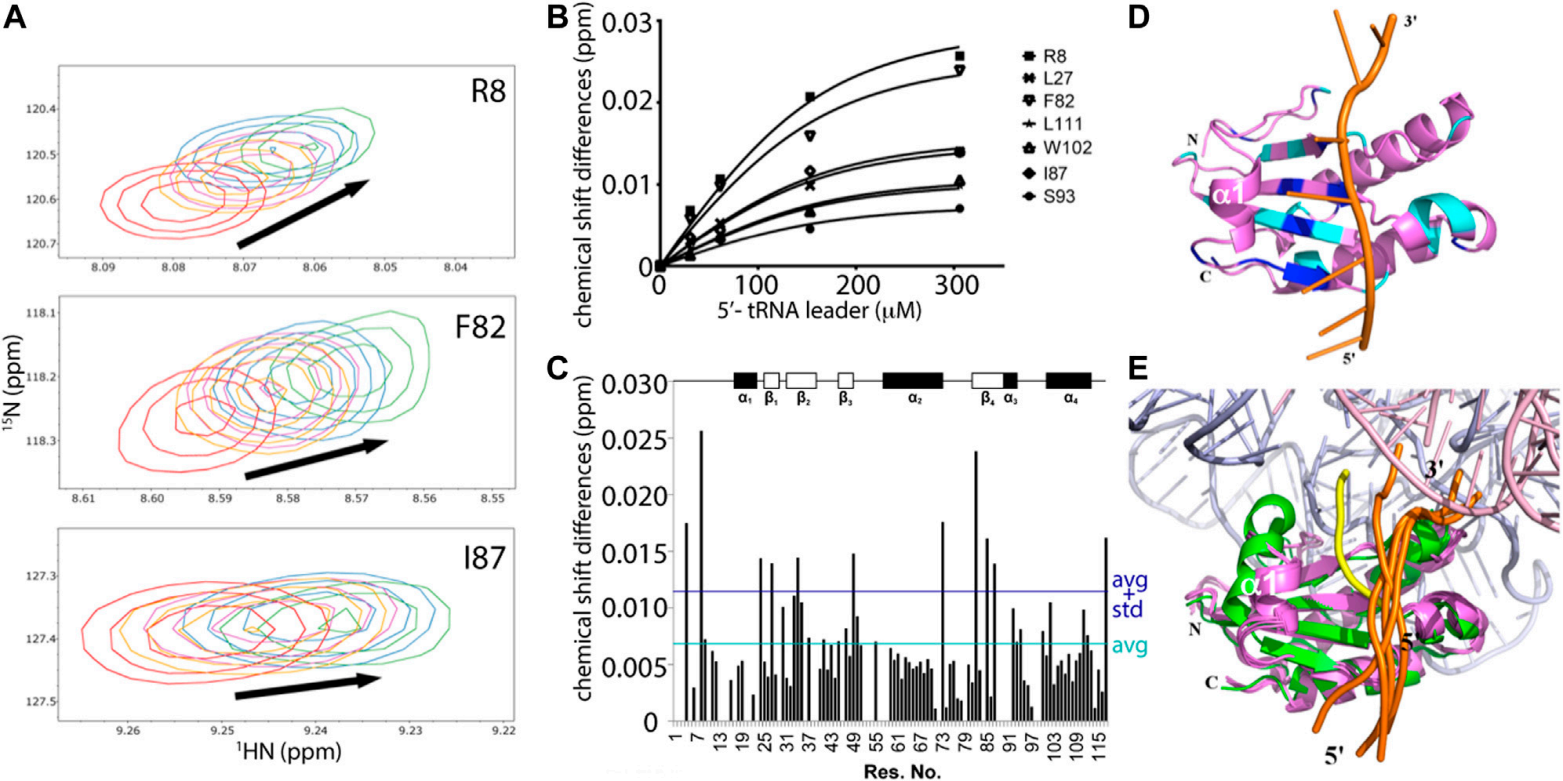
NMR mapping and modeling of the P protein-5’ pre-tRNA leader interaction. **(A)** Representative chemical shift perturbations (CSP) corresponding to RNA titrations to a 153 μM P protein sample. Overlay of ^1^H, ^15^N-HSQC spectra show the chemical shift changes due to RNA binding, where RNA-protein ratios are 0:1 (red), 0.2:1 (orange), 0.4:1 (purple), 1:1 (blue), and 2:1 (green). **(B)** Example curves included in global fitting of deriving dissociation constant of *T. maritima* P protein-5’ leader interaction (K_D_ = 47.2 ± 14.0 μM, *R* ^2^ = 0.983). **(C)** CSP map of ligand-protein molar ratio at 2:1, with indicated chemical shift changes (∆δ) (*y*-axis) by residue (*x*-axis). The dark blue line represents one standard deviation above the average chemical shift difference (∆δ_average_ + STD_∆δ_). The cyan line represents the average chemical shift difference (∆δ_average)._ Schematic plot shows the P protein secondary structure (top). **(D)** The best scored HADDOCK model of the P protein-5’ leader interaction in solution. The lowest energy NMR-derived CS-ROSETTA model (violet) is depicted as ribbons (helix) and arrows (β-sheets) and the 5’ leader RNA is shown as a cartoon (orange backbone trace and green/blue nucleobases). Residues with significant CSP (Δδ ≥ ∆δ_average_ + STD_∆δ_) are highlighted in dark blue, and residues with large CSP (∆δ_average_ + STD_∆δ_ ≥ Δδ ≥ ∆δ_average_) are colored in cyan. The CS-Rosetta generated model derived from monomeric chemical shift data was used in HADDOCK calculations to compare NMR-based data in solution to the available X-ray data (PDBs 1NZ0 and 3Q1R). **(E)** A structural overlay of the HADDOCK generated models (protein/5’ leader, colored as violet/orange) in the context of the bacterial RNase P holoenzyme crystal structure, which emphasizes the P protein-5’ leader interface, colored green/yellow, that is derived from electron density data (PDB 3Q1R). 4 HADDOCK models with the lowest energies were superposed with PDB 3Q1R. The RNase P RNA and tRNA are colored light blue and pink, respectively, and the 5’ and 3’ ends of the leader RNAs are labeled.

Nonetheless, the magnitude of changes in peak positions induced by RNA binding correlate to weak protein-RNA interactions that occur in fast exchange on the NMR timescale (Figure 5A). The dissociation constant (K_D_) was determined by examining the individual change in peak positions (Δδs) against ligand concentrations for residues attributed to only the monomer P protein. 16 data sets were fitted and then combined for a global fitting K_D_ determination, yielding an overall apparent K_D_^global^ of 47.2 ± 14.0 μM (Figure 5B). Significant chemical shifts were observed exclusively in the identified monomer assignments (11 residues, S4, R8, G24, L27, V34, V49, R73, ^82^F, V85, I87, and G117), where significant shifts imply Δδ ≥ ∆δ_average_ + STD_∆δ_ (Figure 5C). Nearly all mapped protein residues undergo fast exchange kinetics upon RNA binding except for amide residues of S26 and V99, which are selectively line broadened and undergo millisecond exchange kinetics. The observed different binding modes may be due to the 5’ leader RNA interactions with the monomer and a heterogeneous monomer-dimer species, thus complicating interpretation. For this reason, only residues that have defined monomeric chemical shift assignments and undergoing fast exchange were included in the dissociation constant calculation. The distributed RNA binding P protein residues were mapped on one of the CS-Rosetta derived model and, as expected, residues with the largest changes in peak positions are located across the β-sheet binding cleft (Figure 5D). These NMR titration experiments validate the 5’ leader-binding interface previously identified in the *T. maritima* RNase P holoenzyme-tRNA complex (Reiter et al., 2010).

Based on CSP results, HADDOCK was performed to generate an ensemble of the P protein monomer-5’ pre-tRNA leader interaction in solution (Figures 5D,E). Chemical shift perturbation results were included as ambiguous restraints between the individual P protein residues of the 5’ leader RNA. Monomeric P protein residues exhibiting peak changes located near the central β-sheet cleft were selected for docking, consistent with *T. maritima* RNase P structure and biochemical studies (Reiter et al., 2010; Reiter et al., 2012). Models generated were solely dependent upon CSP data and emphasize the flexibility of the P protein in accommodating a broad array of 5’ leader RNA nucleotides.

## Discussion

Sample heterogeneity, oligomerization, and the identification of a functionally relevant conformer represent long-standing challenges in the analysis of biochemical data. Dimerization and higher order oligomers of RNA binding proteins can function as essential features for splicing regulation, post-transcriptional processing, and RNA biogenesis, or they can represent aberrant pathways prone to aggregation that can dominate the pathology of a disease (Lagier-Tourenne et al., 2010; Couthouis et al., 2011; Prusty et al., 2017; Montalbano et al., 2020; Montemayor et al., 2020). In bacterial RNase P, the issue of a functional dimeric holoenzyme has been extensively discussed (Fang et al., 2001; Barrera et al., 2002; Barrera and Pan 2004; Buck et al., 2005a; Niland et al., 2017), though it has been structurally validated that the RNA-protein holoenzyme complex in bacteria and eukaryotes function as single, monomeric assemblies to perform ptRNA recognition and catalysis. The propensity for dimer formation is apparent in some bacteria P RNA and P protein crystal structures, potentially masking a functionally relevant conformation in solution (Kazantsev et al., 2003; Torres-Larios et al., 2005). Specifically, the oligomeric state of the P protein crystal structures can block the 5’ leader RNA binding interface, making it difficult to obtain atomic level insight into the RNA binding interface of the bacterial RNase P holoenzyme.

Here, we developed an NMR-based method to overcome oligomerization of the P protein from *Thermotoga maritima* and investigated the RNA binding interface in solution. Diffusion coefficient and NMR relaxation experiments independently validate a concentration-dependent monomeric P protein in solution. In addition, the determination of distinct chemical shift assignments for the monomer and the dimer conformations indicate that monomer-dimer equilibrium undergoes slow exchange kinetics on the NMR timescale. A comparison of the CS-Rosetta predicted structures from chemical shifts from the monomer show that the N-terminus of the P protein has increased flexibility in the absence of RNA ligand or dimer formation, with residues 14–17 no longer part of a stable helix and lacking structural convergence. This conformational flexibility within the N-terminus is consistent with previous NMR relaxation studies of the P protein (Spitzfaden et al., 2000; Henkels et al., 2007). This suggests that the N-terminus (residues 1–17) represents a disordered region of the *T. maritima* P protein in the absence of P RNA or the ptRNA substrate.

**FIGURE 6:**
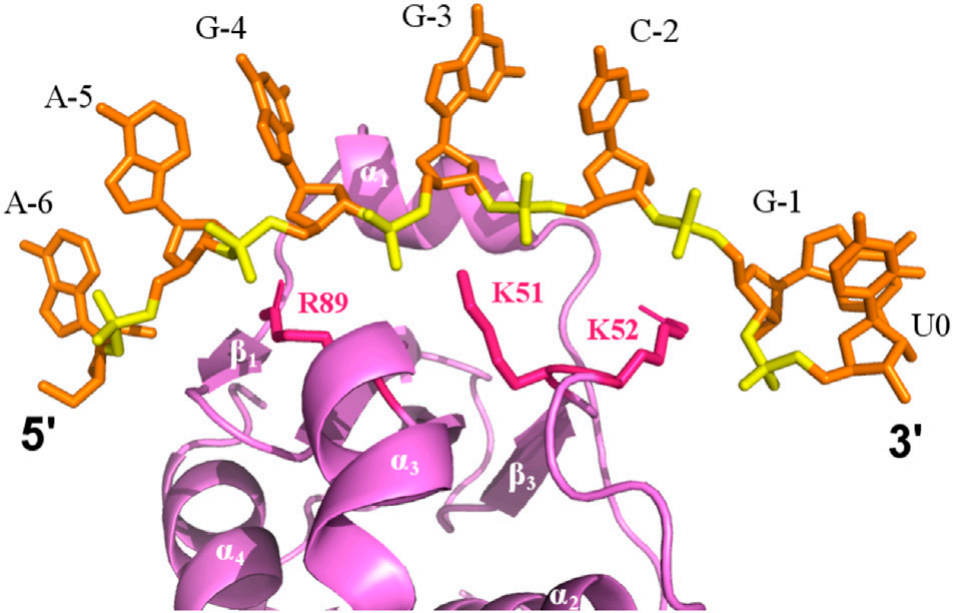
Electrostatic binding interface of a P protein–5’ leader complex in solution. The predicted structure is the best-scored model calculated by HADDOCK. The 5’ leader (orange sticks) lies along the β-sheet cleft of P protein (violet). Electrostatic interactions between positive charged residues of the P protein (sticks, pink) are labeled and are positioned close to the phosphate groups of the 5’ leader (yellow).

In addition, NMR-monitored titration of a short 7-mer 5’ leader RNA show that the largest changes in peak positions due to RNA binding occur across the β-sheet binding cleft that correspond to the 5’ leader binding site previously identified via x-ray crystallography. Structure prediction of the P protein-5’ leader RNA complex based on experimental CSP-NMR analysis indicate that complex formation is largely stabilized through electrostatic interactions between electropositive amino acid side chains and the RNA phosphate backbone (Figure 6). Taken together, and in conjunction with previous structural studies, these results support a model where flexibility of the N-terminus and α1 helix of the P protein can become stabilized through formation of a holoenzyme complex (Henkels et al., 2007; Reiter et al., 2010; Reiter et al., 2012). This P protein N-terminus flexibility may not only be important during holoenzyme assembly and promoting optimal alignment of the 5’ leader of the pre-tRNA substrate, but may also contribute to product release of the cleaved tRNA product.

Due to the large size of the functional enzyme and potential sample heterogeneity in some RNase P systems, few solution NMR studies exist of RNase P components (Schmitz and Tinoco 2000; Spitzfaden et al., 2000; Getz et al., 2007; Henkels et al., 2007; Koutmou et al., 2010a; Zeng et al., 2018). Our NMR-based study provides an avenue for dissecting the RNA interactions of a distinguishable monomeric P protein in solution; however, there are some limitations of this approach. Additional insight into the conformational changes of the structure and P protein-5’ leader interface could be gleaned through pH-titration experiments, the addition of osmolytes, the alteration of salt concentrations, and the application of high pressure NMR. All of these sample optimization strategies could be effective in controlling the oligomeric state of the P protein or RNase P holoenzyme, and it is possible that the observed dimer interactions are unique to the *T. maritima* P protein (Supplementary Figure S4, S5). Nonetheless, the ability to distinguish between monomer and dimer conformations demonstrates the utility of NMR and provides an excellent starting point for additional sample optimization, helping to further shift the equilibrium completely towards a monomeric P protein conformation. Another limitation of the present NMR study is that only amide chemical shift changes were monitored in NMR relaxation studies and to map the RNA binding site, yet it appears that protein side chain flexibility is critical to understand RNA binding specificity, with side chain electrostatic stabilization occurring along the β-sheet binding cleft and potential hydrophobic interactions with residues within the α1 helix. Protein side chain-RNA interactions through CSP analysis or through intermolecular protein-RNA NOE experiments would provide atomic-level insight into the potential nucleobase binding specificity within the 5’ leader RNA region.

Through combined chemical shift perturbation analysis and NMR-based structure prediction studies, we have illuminated the N-terminal structural flexibility of a bacterial P protein in solution and validated the 5’ leader RNA binding interface. Consistent with previous structural and biochemical studies, we confirm that a series of weak, electrostatic-based interactions along the β-sheet binding cleft help to explain how the P protein accommodates different 5’ leader single stranded RNAs and can optimally align a variety of RNA substrates. Dissecting conformational heterogeneity within the N-terminus and monitoring RNA-side chain interactions of the *T. maritima* P protein serve as key next steps towards understanding how intrinsically disordered regions contribute to RNA binding specificity and the activation of the RNase P holoenzyme.

## Data Availability

The datasets presented in this study can be found in the BMRB online repository (accession numbers 27307 and 51021).
