# Supplementary material for: Dissecting Monomer-Dimer Equilibrium of an RNase P Protein Provides Insight Into the Synergistic Flexibility of 5’ Leader Pre-tRNA Recognition
Source: Front Mol Biosci. 2021 Sep 3;8:730274. doi: 10.3389/fmolb.2021.730274 (PMC8447495; doi:10.3389/fmolb.2021.730274)
Supplement: Supplementary file 1 [file DataSheet1.pdf]

Supplementary Material for

Dissecting monomer-dimer equilibrium of an RNase P protein provides insight into the synergistic flexibility of 5' leader pre-tRNA recognition.

Danyun Zeng [1], Ainur Abzhanova [1], Benjamin P. Brown [2,3], and Nicholas J. Reiter [1,*]

[1] Department of Chemistry, Marquette University, Milwaukee, WI 53233, USA

[2] Chemical and Physical Biology Program, Medical Scientist Training Program, Vanderbilt University, Nashville, TN 37205, USA

[3] Center for Structural Biology, Vanderbilt University School of Medicine, Nashville, TN 37205, USA

Supplemental Table 1. Residues with distinct chemical shift assignments in the 2 conformations

	Defined and distinct chemical shift assignments (chemical shift difference > half width of the peak)
Residue No.	8 9 10[1] 11 18[1] 19[2] 21[1] 22 24[2] 25 26 27 28 33 34 35[1] 37[1] 40[3] 43 44 47 48 49 50 51 55 59 60 62 63 64 66 75 82 83 85 86[1] 87 93 94 95 96 97 100 101 102 107 108

[1] The resonance at 153 μM P protein concentration is not observable.
[2] The resonance at 153 μM P protein concentration overlaps with peaks of other residues.
[3] The resonance at 466 μM P protein concentration overlaps with peaks of other residues.

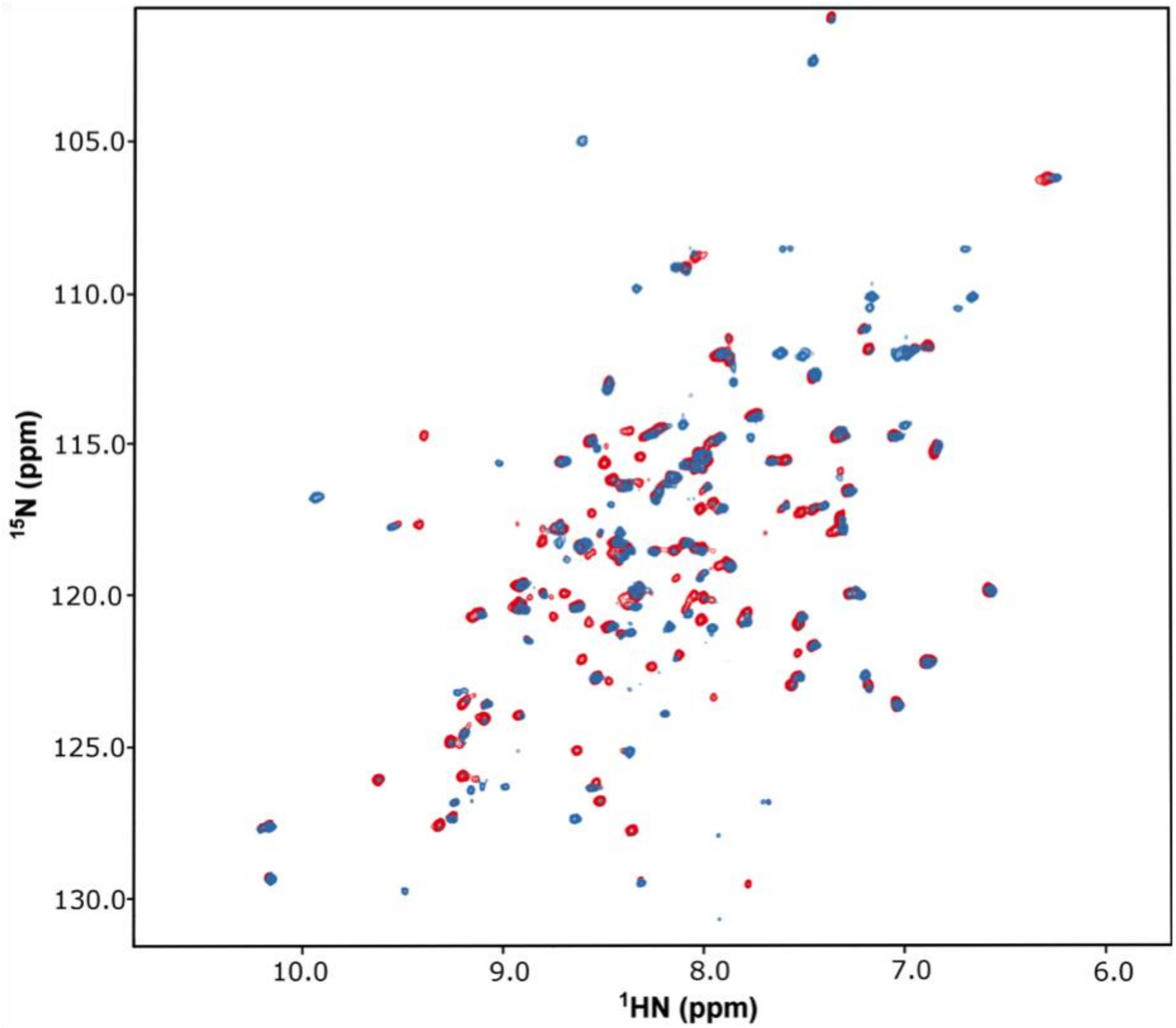

Supplemental Figure 1. Overlay of $^{1}H,^{15}N$-HSQC spectra of *T. maritima* P protein at high (466 μM) and low (153 μM) concentrations at 318 K. Spectra are overlapped by chemical shifts, with high (red) and low (blue) concentrations identified. Contour levels are rendered such that peaks of non-dominant species are observable. Slow exchange kinetics between the monomer-dimer equilibrium reveals distinct chemical shifts and potential conformational difference of P protein at high and low concentrations. A total of 48 residues were confirmed to contain distinct chemical shifts at high and low protein concentrations (listed in Supplement Table 1).

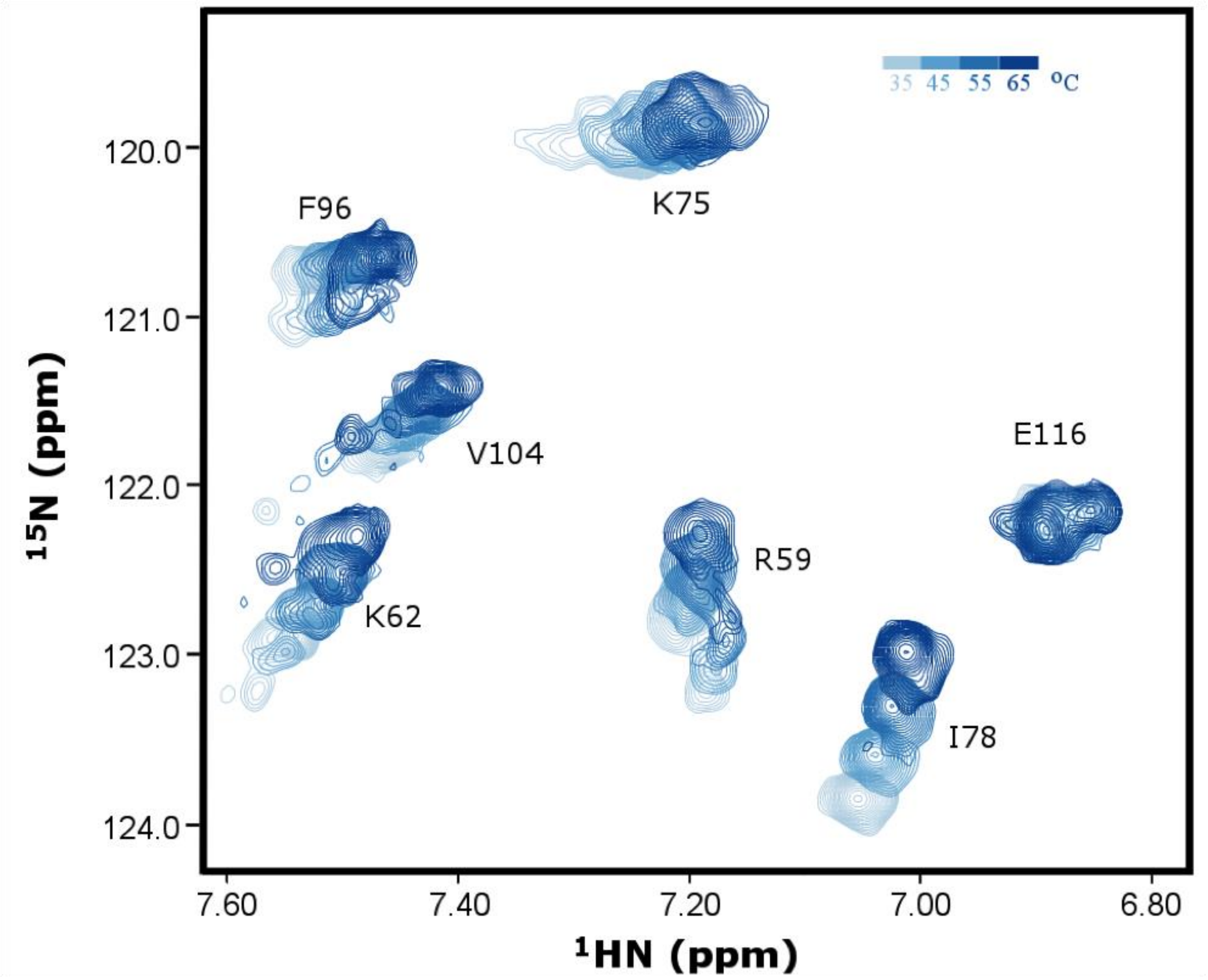

Supplemental Figure 2. Temperature-dependent ^{1}H,^{15}N-HSQC spectra of *T. maritima* P protein at 153 μM. Spectra were acquired at 35 °C, 45 °C, 55 °C and 65 °C (light to deep blue gradient, respectively). At 153 μM, the annotated resonances represent the dominant monomeric conformer, while the dimer conformer is represented by the secondary, very weak peak intensities. As the temperature increases, all peaks undergo uniform chemical shift changes and no obvious peak intensity changes occur. This suggests the monomer-dimer equilibrium in solution is largely independent of temperature.

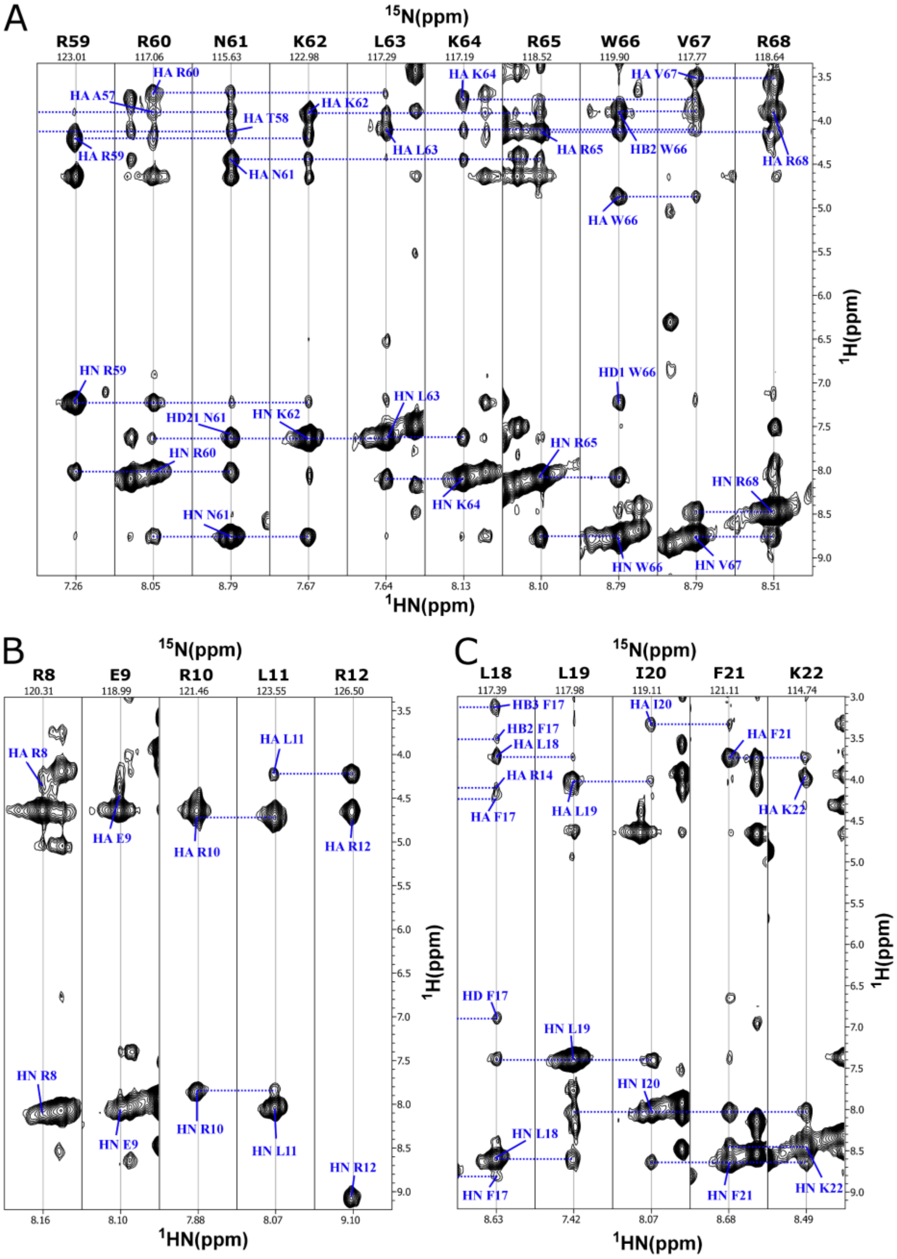

Supplemental Figure 3. Sequential NOE connectivities of the *T. maritima* RNase P protein reveal the extent of secondary structure in solution. Strip plots from the analyzed 3D ^{15}N-NOESY HSQC spectrum include amino acid regions R59-R68 (α_3 helix) (**A**), residues R8-R12 (random coil) (**B**), and residues L18-K22 (α_1 helix) (**C**). The x-axis is the proton dimension of the amide (HN) resonance and the y-axis is the proton dimension that includes Hα – HN NOEs. The strip plots were assigned via one-letter amino acid codes. The 3D ^{15}N-NOESY HSQC spectrum was collected at 318 K with a 120 ms mixing time.

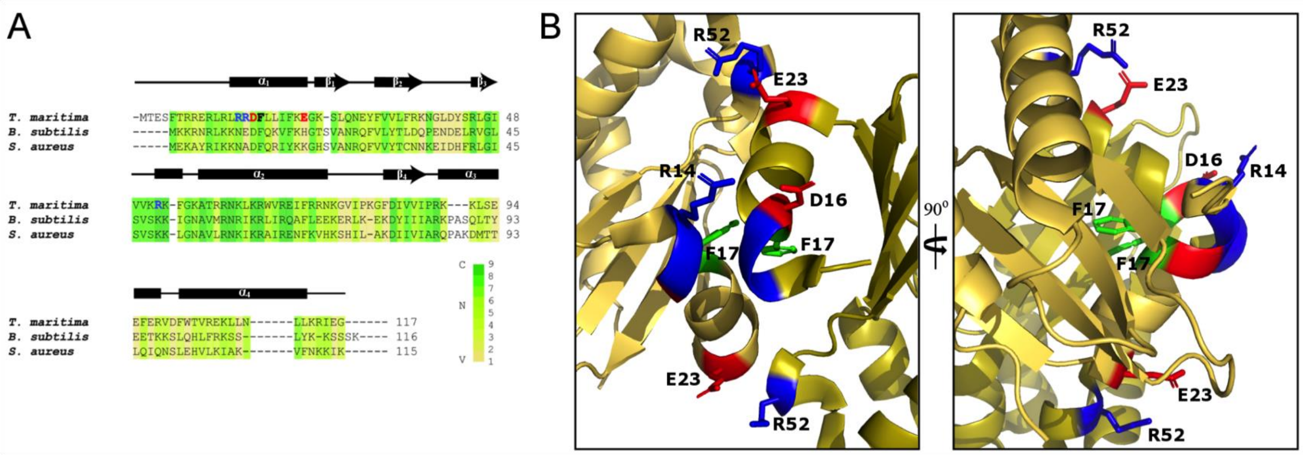

Supplemental Figure 4. N-terminal residues promote stable dimerization of *T. maritima* RNase P proteins. **A.** Sequence alignment of the RNase P protein of *T. maritima*, *B. subtilis* and *S. aureus*. In the sequence of *T. maritima*, bold amino acids are residues involved in dimerization, with positively charged (blue), negatively charged (red), and hydrophobic (black) residues. Secondary structure elements are shown on top. Sequences include: *T. maritima* (gi:9789801), *B. subtilis* (gi: 585905) and *S. aureus* (gi:296275688). Sequences are colored by sequence conservation on a scale of 1 to 9 (1: Variable: tan, Neutral: light green, 9: Conserved: green), as defined previously (Reiter et. al, Nature 2010). Four hundred and ninety bacterial RNase P proteins were included in the analysis of sequence conservation using the ConSurf server17, adapted from (Reiter et al, Nature 2010). **B.** The dimerization interface of *T. maritima* RNase P protein (PDB ID 1NZ0). The conformational difference of monomer and dimer derived CS-Rosetta models shed light on the origins of dimerization observed in the *T. maritima* P protein. Two arginine of residue 14 and 15 with positive charges reside in the α1 helix. These residues are not well conserved in all bacterial P proteins (**A**). In the dimer, R14 interacts with D16 from a different molecule, forming a salt bridge. In addition, E23 and R52 form an electrostatic interactions along the α1 helix. These specific dimer contacts in *T. maritima* P protein likely help to strengthen a hydrophobic stacking interaction of conserved F17 residues. These residues occlude 5' leader RNA binding when a stable dimer is formed. Left and right panels show orthogonal views of the interface from P protein molecules (olive and dark olive cartoons). Residues that contribute to dimerization are highlighted: charged residues providing electrostatic stabilization are blue (positive) or red (negative), while hydrophobic residues forming aromatic stacking interactions are green. Side chain contacts (sticks) emphasize the intermolecular dimer contacts.

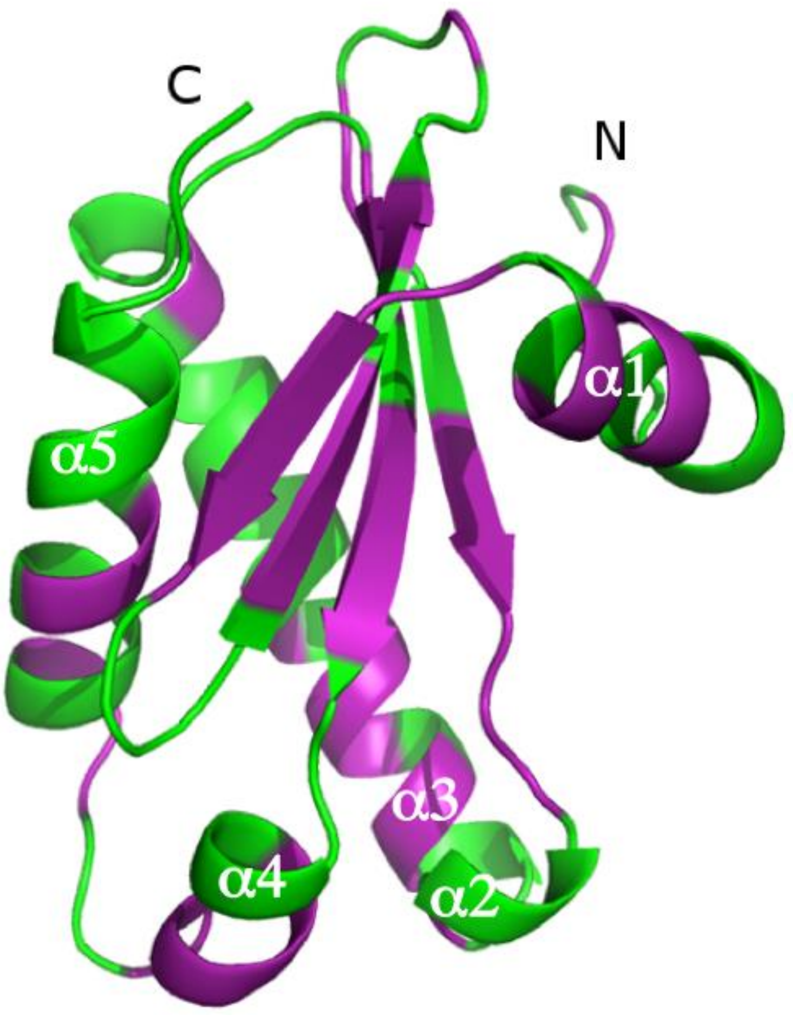

Supplemental Figure 5: Mapping residues that contatin different chemical shifts due to monomer-dimer P protein equilibrium. The residues listed in supplement table 1 contain distinct chemical shifts for the monomer and dimer conformers are highlighted in purple on the crystal structure of P protein (PDB:1NZ0).

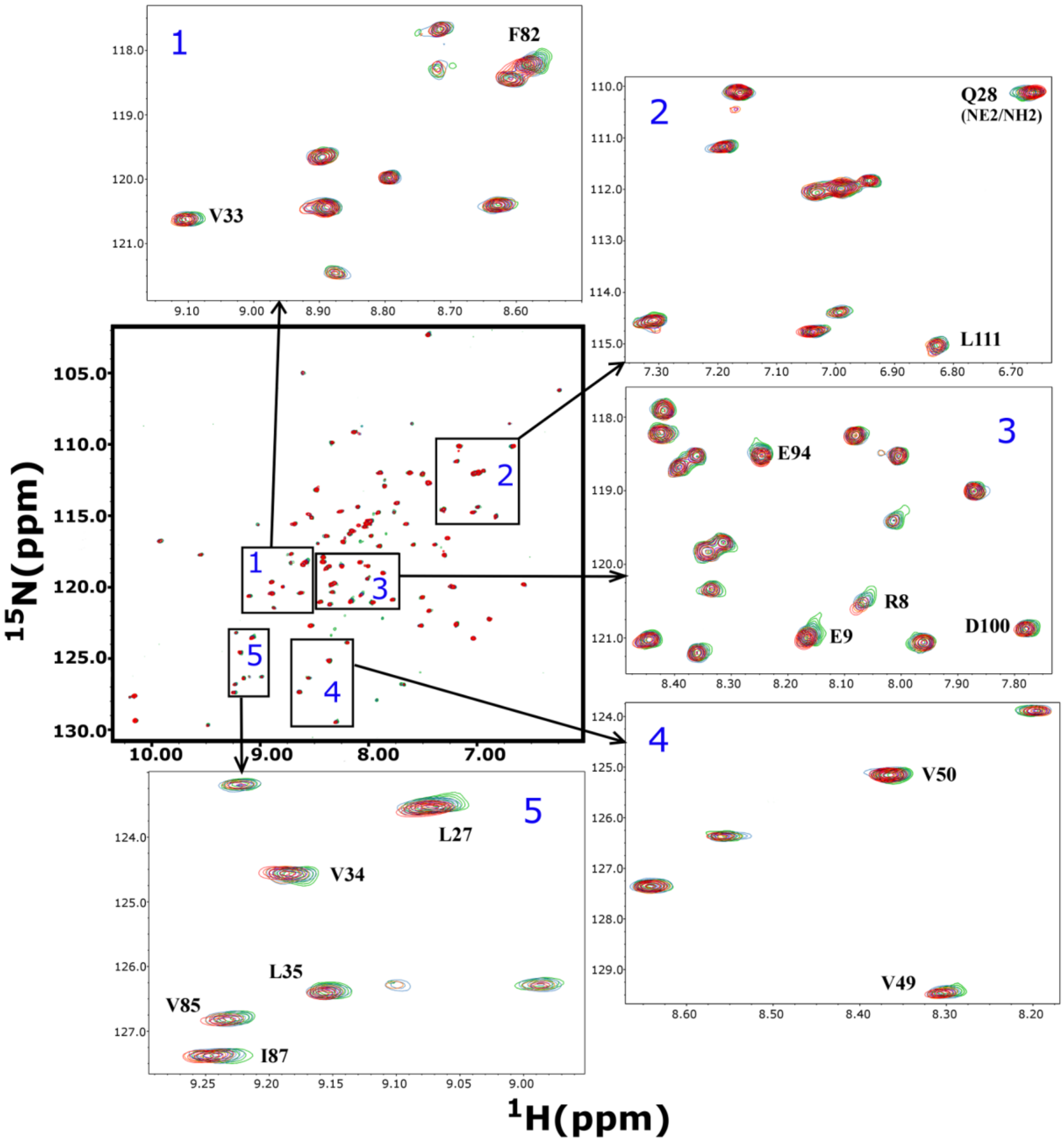

Supplemental Figure 6. Single-stranded RNA binding to the *T. maritima* RNase P protein. The ^{15}N HSQC of the 153 µM *T. maritima* RNase P protein is shown (central) and clockwise (1-5) are zoomed-in regions of the spectra to gauge residues that shift or are unchanged due to RNA binding. During titrations, the leader RNA was gradually added with increased molar ratios of 0:1 (red), 0.2:1 (orange), 0.4:1 (purple), 1:1 (blue), and 2:1 (green) to the 153 µM protein sample. Each region contains labeled single-letter amino acid residues that are influenced by the titration experiment.

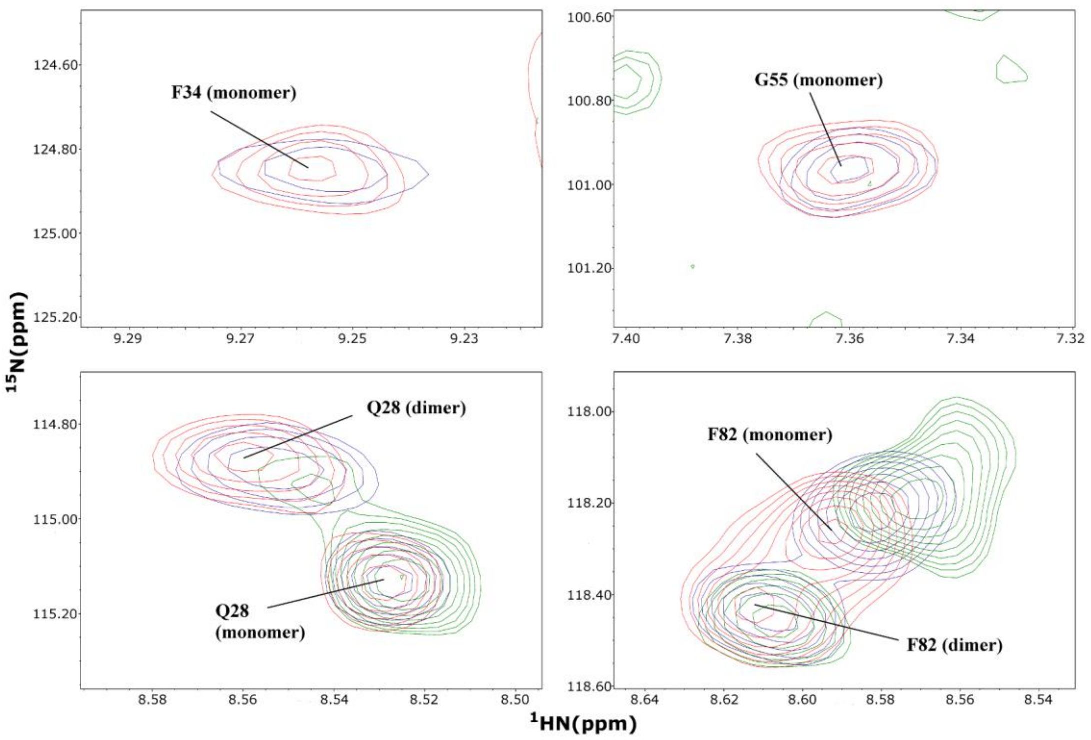

Supplemental Figure 7. Single-stranded RNA binding to the 153 μM *T. maritima* RNase P protein reveals some dimer shifted peaks and unchanged monomer peaks. Examples of dimer conformer peaks **(**Q28 and F82) that shift upon RNA addition, as well as monomer conformer peaks (F34 and G55) that do not shift upon RNA addition. The leader RNA was gradually added at increased molar ratios of 0:1 (red), 0.4:1 (purple), and 2:1 (green) to the 153 μM protein sample. For the Q28 dimer peak, it is possible that RNA addition may shift equilibrium towards the Q28 monomer. For the F82 dimer peak, RNA addition shifts the dimer peak away from a monomer F82 peak and likely does not shift equilibrium towards the monomer conformation. Additional experiments would be required to determine whether specific RNA interactions with the dimer P protein shifts the equilibrium towards the monomeric conformation.
